# Late-Pregnancy Dysglycemia After Negative Testing for Gestational Diabetes and Risk of the Large-for-Gestational-Age Newborns: A Nest Case-Control Study Based on the Xi'an Longitudinal Mother-Child Cohort Study

**DOI:** 10.3389/fped.2022.829706

**Published:** 2022-05-17

**Authors:** Zhangya He, Yang Mi, Hexiang Yang, Jing Ji, Jiayi Gao, Wanyu Zhang, Pei Wu, Zhangrui Xu, Shanshan Wang, Xiaoqin Luo

**Affiliations:** ^1^Department of Nutrition and Food Safety, School of Public Health, Xi'an Jiaotong University, Xi'an, China; ^2^Key Laboratory for Disease Prevention and Control and Health Promotion of Shaanxi Province, Xi'an, China; ^3^Department of Obstetrics and Gynecology, Northwest Women's and Children's Hospital, Xi'an, China; ^4^Emergency Medical Center, Xi'an Public Health Center, Xi'an, China; ^5^Department of Shaanxi Health Supervision Center, Xi'an, China

**Keywords:** GDM, dysglycemia, cohort, obesity, children

## Abstract

**Background:**

The implications of gestational diabetes mellitus (GDM) on the short- and long-term health outcomes of both mother and child have been extensively studied. However, studies related to negative Oral Glucose Tolerance Test (OGTT) results in the second trimester but dysglycemia in late pregnancy on maternal and infant pregnancy outcomes are rare.

**Methods:**

We conducted a nest case-control study within the Xi'an Longitudinal Mother-Child Cohort study (XAMC) to investigate the risk of adverse pregnancy outcomes in mothers and children with maternal negative mid-pregnancy OGTT results but high glycated hemoglobin (HbA_1c_) levels (≥5.7%) in late pregnancy. All target women who delivered from January 1st, 2017 to December 31st, 2018 in Northwest Women's and Children's Hospital in Xi'an were enrolled as the case group (HbA_1c_ ≥ 5.7%). Others with HbA_1c_ < 5.7% but without GDM were selected as the control group (HbA_1c_ < 5.7%) by matching with the same delivery period. The logistic regression models were used to find out the risk factors of adverse pregnancy outcomes in the target population.

**Results:**

A total of 2,116 and 1,907 women were finally enrolled in the case and control groups, respectively. Compared to the control group, more newborns with macrosomia (9.2% vs 4.1%, *P* < 0.001) and large for gestational age (LGA) (23.7% vs. 13.5%, *P* < 0.001), but less small for gestational age (SGA) (4.4% vs. 6.1%, *P* = 0.017) were found in the case group. The differences in other outcomes were not statistically significant. The multiple logistic regression analysis showed that gestational age, fetal length, prenatal HbA_1c_, and total cholesterol (TG) were independent risk factors for newborns with large-for-gestational-age (LGA). The case group had a 2.516-fold (95% CI, 1.692–3.739) risk of delivering LGA newborns compared to the control group.

**Conclusion:**

The glycemic management during the late pregnancy of non-GDM women should be given special consideration to reduce the risk of overweight offspring at birth.

## Introduction

The prevalence of Gestational diabetes mellitus (GDM) varies from 1 to 20% worldwide and is as high as 14.8% in China (1). Moreover, with the implementation of the comprehensive two-child policy, there has been an increasing trend in recent years. GDM is a common metabolic disease during pregnancy, which increases the risk of short- and long-term complications in pregnant women including pre-eclampsia, cesarean section, miscarriage, and diabetes later in life (2–7). With specific clinical diagnosis and treatment guidelines, GDM is receiving extensive attention from doctors, patients, and society.

Notably, recent studies suggested that the presence of relevant dysglycemia in late pregnancy was associated with a higher rate of macrosomia in their offspring at birth and may contribute to the development of childhood overweight and maternal diabetes in obese mothers with negative Oral Glucose Tolerance Test (OGTT) results (non-GDM) (8, 9). Compared to GDM patients, non-GDM women seem to have a clue of an “all-clear signal,” resulting in reduced attention of doctors and a false sense of security in the mothers. A previous study showed the prevalence of high glycated hemoglobin (HbA_1c_) levels (≥5.7%) at delivery was about one out of three in non-GDM women in obese pregnant women (9, 10). Although blood glucose is monitored regularly in late pregnancy, few effective measures are taken to control and treat if dysglycemia happened. In addition to the fact that late pregnancy is a period of significant weight gain for pregnant women, making this special group of pregnancies is more prone to metabolic disorders.

Except for the adverse pregnancy outcomes of the mothers, the short- and long-term effects, such as LGA and childhood obesity, of the disorder of blood glucose metabolism during pregnancy on the offspring are also attracting more and more attention from researchers. Epidemically, over 42 million children (<5 years) worldwide were estimated to be overweight or obese (11). A study has pointed out that the high prevalence of childhood obesity and metabolic diseases in adults was closely related to the child's weight status at birth (12). Since the theory of “fetal programming” suggests that adipose tissue and its secretions, mostly influenced by maternal nutrition/malnutrition and dysglycemia during pregnancy, have direct or indirect effects on childhood obesity (13), potentially modifiable factors in the relationship between offsprings childhood overweight and maternal glucose homeostasis are worth exploring.

Therefore, to find out the risk of offspring overweight at birth in non-GDM women with dysglycemia in late pregnancy, we conducted a nest case-control study in the Xi'an longitudinal mother-child cohort study (XAMC) by using a unique set of longitudinal data of mothers and their newborns. The finding of our study is of particular relevance to proposing effective intervention and prevention measures in the target population and would provide more evidence for blood glucose management throughout pregnancy.

## Methods

### Study Population

XAMC is an ongoing prospective cohort study designed to investigate the effects of early intrauterine exposure on mothers and their offspring outcomes in Xi'an, China. Briefly, XAMC recruited women during early pregnancy at Northwest Women's and Children's Hospital (NWCH), the most extensive obstetrics and gynecology hospital in northwestern China, with 20,000–30,000 deliveries per year (14). In this study, all pregnant women with negative OGTT results in the second trimester who delivered from January 1st, 2017 to December 31st, 2018 were selected as the study population. Women who had a singleton live birth with over 28 weeks gestational age, and had GDM testing data (75-g OGTT according to the IADPSG recommendation) (15) between 24 and 28 weeks of gestation and HbA_1c_ data during the late pregnancy were eligible to be included in the analysis. The detailes of sample size calculation process was provided in the Supplementary Material. The study was approved by both the Research Ethics Committees of NWCH (NWCH2012-013) and Xi'an Jiaotong University (XJTU2016-053). Written consent was obtained from participants.

### Grouping

Blood glucose concentrations were obtained from the hospital medical data system. According to the hospital's usual protocol of assessment of blood glucose in antenatal periods, all pregnant women who met the inclusion criteria had performed an OGTT test (including FPG) in mid-pregnancy (24–28 weeks) and had an HbA_1c_ test at delivery. Details of the OGTT and HbA_1c_ testing are available in the previously reported literature (8). Women with negative OGTT results were assigned to two groups according to the HbA_1c_ levels during late pregnancy. The case group had a sign of dysglycemia with HbA_1c_ ≥ 5.7%. Others with HbA_1c_ < 5.7% matched with the delivery period of the case group were selected as the control group (9). GDM testing was defined as negative when none, and positive when 1 or more, of the 3 glucose concentrations of a 75-g OGTT met or exceeded the reference values according to the IADPSG criteria: fasting glucose ≥ 5.1 mmol/l, 1-h post-load glucose ≥ 10 mmol/l, or 2-h post-load glucose ≥ 8.5 mmol/l (15). The term “late-pregnancy dysglycemia” was used when the maternal HbA_1c_ value during the late pregnancy was greater than or equal to the cut-off of 5.7% (39 mmol/mol) (8).

### Outcomes Variables

Maternal pregnancy outcomes included lateral episiotomy, cesarean section, and postpartum bleeding. Information on maternal primiparity, history of adverse pregnancy, education level, family history of the disease, complications during pregnancy, maternal HbA_1c_ in late pregnancy were extracted from the mothers' pregnancy records. Pre-conception BMI was reported by the woman at this first visit and documented in the pregnancy record booklet. Total gestational weight gain (GWG) in pregnancy was defined as the difference between the weight at delivery and pre-conception weight. The child's characteristics, such as sex, length, weight, gestational age, APGAR score were collected at birth. Macrosomia defined as birth weight ≥ 4,000 g and large-for-gestational-age (LGA) defined as birth weight >90th percentile of the mean weight for the same gestational age (16). Meanwhile, the small-for-gestational-age (SGA) was defined when birth weight was below the 10th percentile of the mean weight for the same gestational age.

### Statistical Analysis

Continuous variables were summarized as means ± SDs if normally distributed and medians with IQRs if not normally distributed. Differences in characteristics between the two groups were assessed by a Student's *t*-test (normal distribution) or Mann-Whitney U test (non-normal distribution) for continuous variables, and a *Chi*-square test for categorical variables. Risk factors were analyzed using logistic analysis. Adjustments were made for potential confounding variables, including family history of diabetes, mode of delivery, height, pre-pregnancy weight, weight gain during pregnancy, prenatal weight, gestational age, newborn length, and prenatal triglycerides. Analyses were carried out using SPSS statistical software (version 24) (SPSS Inc., Chicago, USA). Significance was defined as *P* < 0.05 (two-tailed).

## Results

### Basic Characteristics of Participants

**Figure 1:**
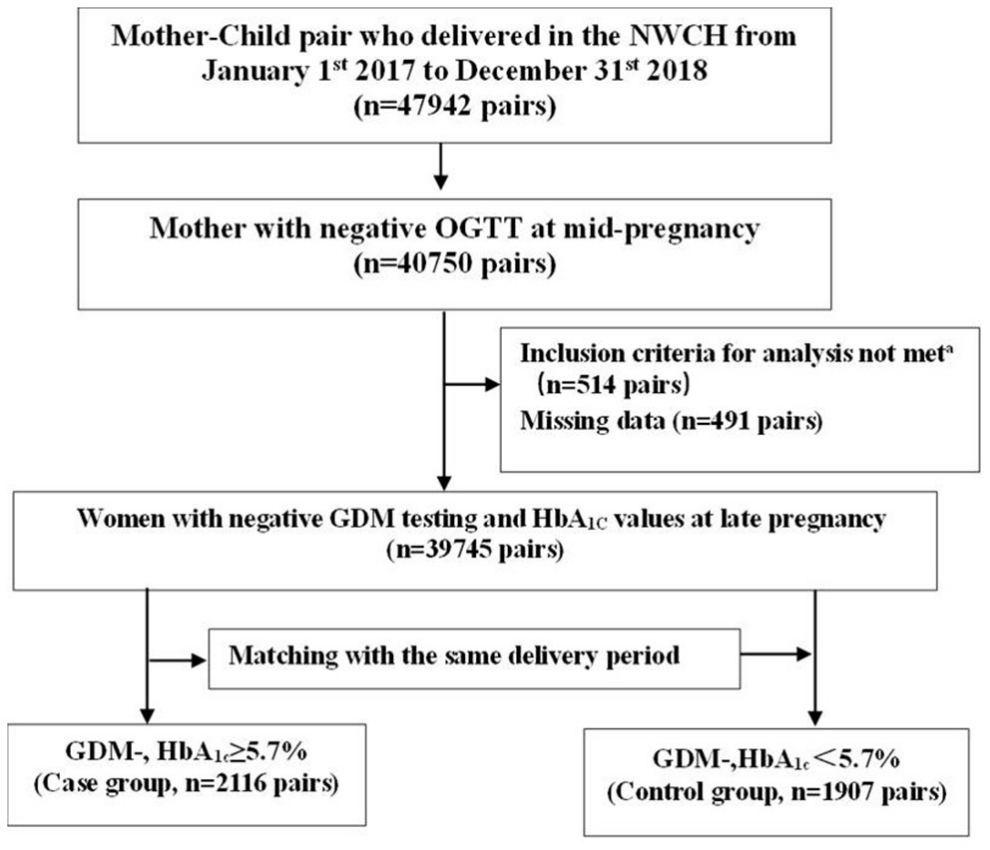
Flow diagram outlining the selection of the study population. Inclusion criteria for analysis include singleton live birth, over 28 weeks gestational age, and absence of type 1 diabetes and type 2 diabetes. missing information for at least 1 of the following variables: pre-conception body mass index group, maternal HbA_1c_ at late pregnancy (<5.7% [39 mmol/mol] or ≥5.7%), or confounding variables. GDM-, gestational diabetes mellitus negative; HbA_1c_, glycated hemoglobin.

There were 47,942 mother-child pairs during the study period and almost 85% (40,750) of them had negative OGTT results at mid-pregnancy. Finally, 39,745 met the inclusion criteria. Among them, there were a total of 2,116 (5.32%) pairs with HbA_1c_ ≥ 5.7% (the case group), and 1,907 pairs with HbA_1c_ < 5.7% (the control group, which matched the same delivery period) (Figure 1). All mothers involved in this study did not develop any obvious symptoms related to diabetes throughout pregnancy, even diabetes requiring insulin therapy or any clinical symptoms of insulin resistance, like acanthosis nigricans. As shown in Table 1, compared to the control group, the case group had a higher advanced age (16.9% vs. 14.4%, *P* = 0.03), a lower education level (13.3% vs. 16.5%, *P* = 0.014), higher proportions of family history of diabetes (7% vs. 4.8%, *P* = 0.014), anemia (51.8% vs. 42.7%, *P* < 0.001) and overweight and obese before pregnancy (13.6% vs. 9%, *P* < 0.001), and more gestational weight gain (GWG). Other characteristics of participants, such as parity, history of adverse pregnancy, other complications during pregnancy and biochemical indicators of lipid metabolism were comparable between the two groups. Moreover, the distribution characteristics of the offspring presented in Table 2 showed that the newborns were heavier (3434.08 ± 440.26 vs. 3322.66 ± 456.72, *P* < 0.001) and had a longer birth length (50.31 ± 0.97 vs. 50.06 ± 2.06, *P* < 0.001) in the case group compared with the control group.

**Table 1:** Basic characteristics of participants according to late pregnancy HbA_1c_ categories.

**Characteristics**	**Case group** ** (*n* = 2,116)**	**Control group** ** (*n* = 1,907)**	***x***^**2**^/***t***	***P***
**Age at conception, years**, ***n*** **(%)**				
≥35	358 (16.9)	275 (14.4)	4.721	0.030
<35	1,758 (83.1)	1,632 (85.6)		
**Education level**, ***n*** **(%)**				
High School and below	282 (13.3)	314 (16.5)	8.503	0.014
College degree	1,500 (70.9)	1,320 (69.2)		
Postgraduate and above	334 (15.8)	273 (14.3)		
**Family history of diabetes**, ***n*** **(%)**	148 (7)	91 (4.8)	8.866	0.003
**Family history of hypertension**, ***n*** **(%)**	320 (15.1)	262 (13.7)	1.553	0.213
**Pre-conception BMI (kg/m**^**2**^**)**, ***n*** **(%)**				
<18.5	220 (10.4)	324 (17)	51.683	<0.001
18.5–24.9	1,609 (76)	1,411 (74)		
25.0–29.9	249 (11.8)	154 (8.1)		
≥30.0	38 (1.8)	18 (0.9)		
Total GWG (kg)^*^				
<18.5	17.00 ± 4.26	15.45 ± 4.18	4.209	<0.001
18.5–24.9	16.16 ± 4.39	15.17 ± 4.09	6.359	<0.001
25.0–29.9	14.47 ± 4.92	12.90 ± 5.06	3.072	0.002
≥30.0	12.03 ± 4.78	11.83 ± 4.00	0.148	0.883
**Parity**, ***n*** **(%)**				
Nulliparous	1,453 (68.7)	1,288 (67.5)	2.037	0.361
Multiparous	663 (31.3)	619 (32.5)		
**History of adverse pregnancy**, ***n*** **(%)**	879 (41.5)	808 (42.4)	0.283	0.594
**Complications during pregnancy**, ***n*** **(%)**				
Abnormal thyroid function	171 (8.1)	152 (8)	0.017	0.897
Anemia	1,097 (51.8)	814 (42.7)	33.736	<0.001
Hypertension	43 (2)	29 (1.5)	1.493	0.222
**Biochemical indicators in late pregnancy birth**				
TC (mmol/mol)	5.76 ± 1.06	5.75 ± 0.92	0.278	0.781
TG (mmol/mol)	3.01 ± 1.06	2.96 ± 1.01	1.447	0.148
HDL (mmol/mol)	1.72 ± 0.32	1.74 ± 0.35	−1.732	0.083
LDL (mmol/mol)	3.01 ± 1.06	3.00 ± 0.63	0.358	0.721
HbA_1c_ level (%)	5.85 ± 0.19	5.11 ± 0.33	85.135	<0.001

*Continuous variables meet the conditions of normality, and categorical variables are expressed as mean ± standard deviation and counts (percentages), Separately. And the t-test and chi-square test were used to analyze the differences between groups. P-values <0.05 were considered statistically significant. GWG, gestational weight gain; TG, Triglycerides; TC, total cholesterol; HDL, High-density lipoprotein; LDL, Low-density lipoprotein; HbA_1c_, glycated hemoglobin*.

* *means total gestational weight gain by pre-conception BMI*.

**Table 2:** Newborn offspring characteristics of participants according to late pregnancy blood glucose among the total participants.

**Characteristics**	**Case group** ** (*n* = 2,116)**	**Control group** ** (*n =* 1,907)**	***x***^**2**^/***t***	***P***
**Sex**				
Male	1,184 (56)	986 (51.7)	9.325	0.009
Female	932 (44)	919 (48.2)		
**Birth weight, g**	3434.08 ± 440.26	3322.66 ± 456.72	7.874	<0.001
**Gestational age, weeks**	39.42 ± 1.47	39.41 ± 1.57	0.289	0.772
**Birth length, cm**	50.31 ± 0.97	50.06 ± 2.06	4.913	<0.001
**Apgar score**				
Apgar-1 min	8.97 ± 0.28	8.96 ± 0.40	1.139	0.255
Apgar-5 min	9.98 ± 0.25	9.96 ± 0.42	1.596	0.111
Apgar-10 min	9.98 ± 0.35	9.97 ± 0.39	0.419	0.675

*Continuous variables meet the conditions of normality and categorical variables are expressed as mean ± standard deviation and counts (percentages), Separately. And the t-test and chi-square test were used to analyze the differences between groups. P-values < 0.05 were considered statistically significant. HbA_1c_, glycated hemoglobin*.

### Associations Between HbA_1C_ Values at Late Pregnancy and Pregnancy Outcomes

As shown in Table 3, the differences of the incidence of adverse pregnancy outcomes for mothers and some of the adverse birth outcomes for children between the two groups were not statistically significant, including lateral episiotomy (7% vs. 4.8%, *P* = 0.068), postpartum bleeding (0.9% vs. 1%, *P* = 0.869), and cesarean section (41.4% vs. 41.2%, *P* = 0.904) for mothers, while birth defects (0.3% vs. 0.1%, *P* = 0.085), prematurity (4.8% vs. 4.9%, *P* = 0.878), perinatal mortality (0.3% vs. 0.3%, *P* = 0.857), and fetal distress (1.9% vs. 2.6%, *P* = 0.145) in the offspring. However, there was a remarkable higher prevalence of LGA (23.7% vs. 15.3%, *P* < 0.001) and macrosomia (9.2% vs. 4.1%, *P* < 0.001) in the offspring of the case group.

**Table 3:** Adverse outcomes of participants and offspring according to late pregnancy blood glucose among the total participants.

**Adverse outcomes**	**Case group** ** (*n =* 2,116)**	**Control group** ** (*n =* 1,907)**	***x***^2^/***t***	***P***
**Maternal outcomes**				
Lateral Episiotomy	453 (21.4)	364 (19.1)	3.338	0.068
Postpartum bleeding	20 (0.9)	19 (1)	0.027	0.869
Cesarean section	875 (41.4)	785 (41.2)	0.015	0.904
**Children outcomes**				
Birth defects	7 (0.3)	1 (0.1)	3.917	0.085
Prematurity	101 (4.8)	93 (4.9)	0.023	0.878
Macrosomia	195 (9.2)	78 (4.1)	41.655	<0.001
Perinatal mortality	6 (0.3)	6 (0.3)	0.033	0.857
SGA	94 (4.4)	117 (6.1)	5.785	0.017
LGA	501 (23.7)	291 (15.3)	44.946	<0.001
Fetal distress	40 (1.9)	49 (2.6)	2.138	0.145

*Categorical variables are expressed as counts (percentages). And the t-test and chi-square test were used to analyze the differences between groups. P-values < 0.05 were considered statistically significant*.

*SGA, small for gestational age; LGA, large for gestational age. HbA_1c_, glycated hemoglobin*.

The univariate logistical analysis with the occurrence of LGA and/or macrosomia as an arbitrary adverse pregnancy outcome (Table 4) showed that HbA_1c_ ≥5.7%, pre-conception BMI < 18.5, prematurity, maternal height, pre-pregnancy weight, GWG, prenatal weight, gestational age, neonatal length at birth, late pregnancy HbA_1c_, and TG were associated with offspring overweight. Further multifactorial logistical analysis indicated that gestational age (OR = 1.644, 95% CI, 1.413–1.914), fetal length (OR = 7.309, 95% CI, 5.774–9.252), prenatal TG (OR = 1.159, 95% CI, 1.019–1.318), and late pregnancy HbA_1c_ (OR = 2.063, 95% CI, 1.077–3.952) were risk factors for offspring overweight. The association between late-pregnancy HbA_1c_ levels and offspring overweight remained after adjusting for family history of diabetes, mode of delivery, height, pre-pregnancy weight, GWG, prenatal weight, gestational age, fetal length, and maternal TG. The risk of offspring being overweight increased to 2.516 (95% CI, 1.692–3.739) times in the case group.

**Table 4:** Univariate and Multivariate analysis of related factors of offspring macrosomia and LGA.

**Variables**	**Univariate analysis**		**Multivariate analysis**		
	**OR (95% CI)**	***P-value***	**Offspring overweight-adjusted Risk Ratio (95% CI)**	**OR (95% CI)**	***P-value***
**Gestational age, week**	1.827 (1.612–2.072)	<0.001	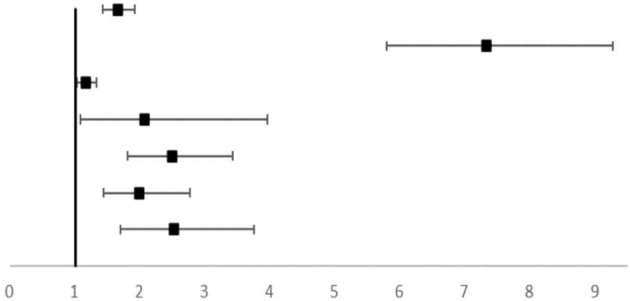	1.644 (1.413–1.914)	<0.001
**Fetal length, cm**	3.828 (3.340–4.387)	<0.001		7.039 (5.774–9.252)	<0.001
**TG (mmol/mol)**	1.214 (1.099–1.340)	<0.001		1.159 (1.019–1.318)	0.025
**Prenatal HbA**_**1c**_	2.479 (1.806–3.402)	<0.001		2.063 (1.077–3.952)	0.029
**Prenatal HbA**_**1c**_ ^**1**^				2.478 (1.801–3.410)	<0.001
**Prenatal HbA**_**1c**_ ^**2**^				1.984 (1.430–2.753)	<0.001
**Prenatal HbA**_**1c**_ ^**3**^				2.516 (1.692–3.739)	<0.001
**Pre-conception BMI (kg/m** ^**2**^ **)**					
<18.5	0.379 (0.218–0.656)	0.001			
18.5–23.9	Ref				
24–27.9	1.406 (0.969–2.0308)	0.072			
≥28	0.811 (0.251–2.619)	0.726			
**Prematurity**	0.074 (0.010–0.534)	0.01			
**Prenatal weight, kg**	1.061 (1.061–2.072)	<0.001			

*A binary Logistic analysis was used to analyze the effect of late pregnancy HbA_1c_ on neonatal birth weight overweight, adjusting for confounding factors such as the family history of diabetes, pre-pregnancy weight, weight gain during pregnancy, and fetal sex and length at birth. TG, Triglycerides; HbA_1c_, glycated hemoglobin; OR, odds ratio; CI, confidence interval*.

1 *Model 1 Adjusted for family history of diabetes, mode of delivery, height*.

2 *Model 2 Adjusted for family history of diabetes, mode of delivery, height, pre-pregnancy weight, weight gain during pregnancy, prenatal weight*.

3 *Model 3 Adjusted for family history of diabetes, mode of delivery, height, pre-pregnancy weight, weight gain during pregnancy, prenatal weight, gestational age, newborn length, prenatal triglycerides*.

## Discussion

In China, the occurrence of dysglycemia in the last trimester of pregnancy despite prior negative testing for GDM and its consequences have not been extensively concerned. Although it would increase the risk of both short- and long-term adverse outcomes for both mothers and children, no guideline for routine monitoring was available clinically. In the present study, we found that intrauterine exposure to late-pregnancy dysglycemia even without GDM is strongly associated with offspring overweight. Our large population-based cohort study provides novel evidence that glycemic management during the late pregnancy of non-GDM women also should be considered to reduce the risk of overweight offspring in early life.

Numerous studies showed that the intrauterine environment impacted early life from conception to the toddler years (the first 1,000 days), even influencing a person's response to later exposures and subsequently modifying their health and disease risk later in life (17–19). Apart from genetic background and lifestyle factors related to maternal obesity, prenatal metabolic influences of a high glucose intrauterine environment seem to play an important role in the offspring being overweight at birth (10, 20). Although the study found that about half of births of women with diabetes were LGA (21), the situation in non-GDM women with glucose metabolism disorders in the third trimester is unclear. A German study recently showed that obese, GDM-negative women with late-pregnancy dysglycemia had higher proportions of offspring with LGA birth weight than those without. Moreover, the offspring of these women had greater weight gain during early childhood (8). Similarly, we also found more women were overweight/obese before conception in that than the target population, and there was a remarkable higher prevalence of LGA (23.7% vs. 15.3%, *P* < 0.001) and macrosomia (9.2% vs. 4.1%, *P* < 0.001) in their offspring. Although the prevalence of late-pregnancy dysglycemia in non-GDM population was around 5% in our study, the absolute number was quite large. Most importantly, the prevalence of macrosomia was 9.2% and LGA was 23.7%. The high prevalence of childhood obesity and metabolic diseases in adults was closely related to the child's weight status at birth (12). LGA neonates have the highest BMI at age 4–7 years (22), putting them at a higher lifetime risk of being overweight and developing obesity and metabolic diseases (23). A negative GDM test at the end of the second trimester was not an “all-clear signal” and did not result in reduced attention from caregivers and a false sense of security in mothers, especially those who are overweight/obese.

It is already widely known that women with GDM not only increased the incidence of stillbirths and birth complications, including a high risk of cesarean delivery, shoulder dystocia, birth injury, and macrosomia (24–26) but also increased the risk of developing T2DM in subsequent years (3–5). But in our study, the difference in adverse pregnancy outcomes between mothers with metabolic disorders in the case and control group was not statistically significant. This may be related to the fact that the population included in our study were non-GDM which indicated that the sustained stability of maternal metabolism in the early and middle stages of pregnancy, the critical period of fetal development, lays a good foundation for a portion of pregnancy outcomes. In addition, our inclusion criteria were only live fetuses older than 28 weeks of gestational age were included, which reduced the possibility of the occurrence of severe adverse pregnancy outcomes such as stillbirths and malformed fetuses. As the cohort progresses successfully, we will continue to monitor and report on the long-term health outcomes of mothers and children.

One of the major strengths of our study was related to the large contemporary mother-child XAMC cohort, which is unique in the size of the population of mothers and the availability of trimester-specific data, including HbA_1c_ at late pregnancy as a marker of glycemic control in late pregnancy. Secondly, this is the first time to find out the risk of offspring overweight in the most neglected group of people with a negative OGTT test in mid-pregnancy but abnormal glucose metabolism in late pregnancy. Despite a negative mid-pregnancy OGTT, the monitoring of maternal glucose throughout the pregnancy cycle should not be ignored; and maintaining glucose throughout the pregnancy cycle has significant public health implications for reducing childhood obesity. The third strength of our study was that outcomes data were ascertained by trained medical staff, and pregnancy and postpartum confounding factors associated with adverse pregnancy outcomes and neonatal macrosomia were comprehensively collected and adjusted for pregnancy complications, pre-pregnancy body mass, and pregnancy weight gain.

However, some limitations of our study have to be considered. Firstly, we only take HbA_1c_ levels into consideration for late pregnancy dysglycemia. Despite a general agreement that maternal late hyperglycemia causing fetal hyperinsulinemia is an important determinant of fetal macrosomia, there is some evidence that the contribution of maternal hyperglycemia to the variance in LGA birth weight is relatively low, and even high rates of LGA birthweight are reported despite apparently normal HbA_1c_ levels (27–29). Measures of glycaemic variation using newer methods, such as continuous glucose monitoring, may contribute to a better understanding of the relationship between hyperglycemia and fetal macrosomia in the future (29). In any case, continuous monitoring of the mother's blood glucose during pregnancy, even those with a negative OGTT test in mid-pregnancy, will reduce the risk of childhood obesity and even metabolic diseases in the future. Secondly, GWG during the 2nd and 3rd trimesters and its correlation with HbA_1c_ will help to understand the dynamics of late trimester dysglycemia on the fetal weight, but this variable was not collected before in this retrospective cohort. However, up to 90% of pregnant women experience nausea and vomiting, and even weight loss in the first trimester (30–32), so the majority of GWG is accounted for by weight gain in the 2nd and 3rd trimesters. And in thanalysis study, we found that even after adjusting for GWG, there still was a relationship between HbA_1c_ levels at birth and LGA newborns (Table 4). Last but not least, this is a cross-sectional presentation of the basic information of an ongoing cohort study, and the causality argument is not strong enough, and further follow-up of long-term health outcomes of mothers and children in this specific population is needed in the future.

## Conclusion

The third-trimester HbA_1c_ levels were the important potentially modifiable predictors of birthweight, reinforcing the need for careful glucose control throughout pregnancy. In future studies, more sensitive measures of both glucose control and fetal growth and body composition should be used to explore critical windows for the effect of maternal blood glucose concentration on birthweight.

## Data Availability Statement

The raw data supporting the conclusions of this article will be made available by the authors, without undue reservation.

## Ethics Statement

The study was approved by both the Research Ethics Committees of NWCH (NWCH2012-013) and Xi'an Jiaotong University (XJTU2016-053). The patients/participants provided their written informed consent to participate in this study.

## Author Contributions

ZH and XL designed the study and wrote the manuscript. ZH and YM performed the analysis and quality control on the data. JG, HY, WZ, PW, JJ, ZX, SW, and YM collected the data. XL had primary responsibility for the final content, is the guarantor of this work and, as such, have full access to all the data in the study and takes responsibility for the integrity of the data and the accuracy of the data analysis. ZH, YM, HY, JG, JJ, WZ, PW, ZX, SW, and XL read and approved the final manuscript and contributed to revising the manuscript critically for important intellectual content. All authors contributed to the article and approved the submitted version.

## Funding

This research was funded by the National Natural Science Foundation of China (No. 81874263) and the Key Special Project of the Development Center for Medical Science & Technology of the National Health Commission of the People's Republic of China (W2015CAE060).

## Conflict of Interest

The authors declare that the research was conducted in the absence of any commercial or financial relationships that could be construed as a potential conflict of interest.

## Publisher's Note

All claims expressed in this article are solely those of the authors and do not necessarily represent those of their affiliated organizations, or those of the publisher, the editors and the reviewers. Any product that may be evaluated in this article, or claim that may be made by its manufacturer, is not guaranteed or endorsed by the publisher.
