# Supplementary material for: Late-Pregnancy Dysglycemia After Negative Testing for Gestational Diabetes and Risk of the Large-for-Gestational-Age Newborns: A Nest Case-Control Study Based on the Xi'an Longitudinal Mother-Child Cohort Study
Source: Front Pediatr. 2022 May 17;10:829706. doi: 10.3389/fped.2022.829706 (PMC9152353; doi:10.3389/fped.2022.829706)
Supplement: Supplementary file 1 [file Table_1.DOCX]

Supplementary Material

# Supplementary Data

**The detailed sample size calculation process is as follows**

This study was derived from a nested case-control study, and according to the requirements of this design type, the sample of this study should be calculated using the cohort study sample size calculation method. The exposed group was women with negative OGTT in mid-pregnancy but abnormal glucose metabolism in late pregnancy and the control group were mothers with normal glucose throughout pregnancy. The occurrence of overweight/obesity in the offspring of the study subjects was the main outcome indicator observed. As reported in the literature ^[1]^, the prevalence of overweight in preschool children in the exposed group was 18.4%, and 12% in the control group. Then setting α=0.05 (bilateral) and β=0.9, N1=655 cases in the exposed group was calculated by using PASS11 software, and approximately 1:1 matching with age and delivery department to obtain N2=655 cases in the control group ^[2]^. Since this study is a presentation of the interim results of a study, the original study design required long-term follow-up of short- and long-term pregnancy outcomes for mother and child and may have faced a high rate of missed visits. Therefore, assuming a 40% lost-to-follow-up rate for study subjects, a sample size of at least N1=917 and N2=917 was required. Finally, 2116 cases were included in the exposure group and 1907 cases in the control group.

**Reference**

[1] Mom's Gestational Diabetes Raises Childhood Risk of Obesity: Worldwide Study. Medscape. Becky McCall August 15, 2016

[2] Chow S, Shao J, Wang H. 2008. *Sample Size Calculations in Clinical Research*. 2nd Ed. Chapman & Hall/CRC Biostatistics Series. page 89.
